# Hand rehabilitation programmes for second to fifth metacarpal fractures: A systematic literature review

**DOI:** 10.4102/sajp.v77i1.1536

**Published:** 2021-05-31

**Authors:** Monique M. Keller, Roline Barnes, Corlia Brandt, Lauren M. Hepworth

**Affiliations:** 1 Department of Physiotherapy, School of Health and Rehabilitation Sciences, University of the Free State, Bloemfontein, South Africa; 2 Department of Physiotherapy, School of Therapeutic Sciences, Faculty of Health Sciences, University of the Witwatersrand, Johannesburg, South Africa; 3 Occupational Therapy, Private practice, Durban, South Africa

**Keywords:** boxer’s fractures, exercises, metacarpal fractures, rehabilitation, therapy

## Abstract

**Background:**

Metacarpal fractures, one of the most prevalent upper limb fractures, account for 10% of all bony injuries.

**Objective:**

Our systematic review aimed to review, appraise and collate available evidence on hand rehabilitation programmes for the management of second to fifth metacarpal fractures in an adult human population after conservative and surgical management. Since 2008, no review on a similar topic has been performed, thus informing clinical practice for physiotherapists and occupational therapists.

**Methods:**

Preferred Reporting Items for Systematic Reviews and Meta-Analysis (PRISMA) principles guided the reporting. Experimental, quasi-experimental, cohort and case–control studies between January 2008 and September 2018 were included. Searches were conducted on Medline, Academic Search Ultimate, CINAHL, CAB Abstracts, Health Source – Consumer Edition, Health Source: Nursing/Academic Edition, SPORTDiscus, Africa-Wide Information and MasterFILE Premier, Web-of-Science and Scopus. Screening, selection, appraisal and data extraction were independently performed by two reviewers. No meta-analysis was performed.

**Results:**

A total of 1015 sources were identified, 525 duplicates removed and 514 excluded. Three articles were included in the final data extraction: one randomised controlled trial (RCT) and two observational studies.

**Conclusion:**

Limited evidence is available that a well-designed, well-implemented home-based exercise programme results in statistically significant improved hand function (*p* ˂ 0.0001) and digital total active motion (TAM) (*p* = 0.013) compared with traditional physiotherapy (PT) post-surgically.

**Clinical implications:**

Our study contributes to the knowledge base of hand rehabilitation after an individual sustained a second to fifth metacarpal fracture. The authors identified a gap where future studies should further investigate the effect of hand rehabilitation after conservative and surgical management.

## Introduction

Metacarpal fractures are amongst the most prevalent upper limb injuries in adults (Bucholz 2009). An incidence rate (IR) for metacarpal fractures is 13.6 per 100 000 with a prevalence of 33% in the United States of America (Nakashian et al. 2012), and boxer’s fractures, break of the fifth metacarpal bones, account for 20% of all hand fractures (Ali, Hamman & Mass 1999). The IR of metacarpal fractures is higher amongst males (IR 28.4) than in females (IR 4.4). Metacarpal fractures frequently occur when the hand makes contact with a solid surface, during falls and in motor vehicle accidents (Nakashian et al. 2012).

As indicated by Cooper and Wietlisbach (2014), hand rehabilitation is important to ensure optimal hand function post-surgery and during conservative management of second to fifth metacarpal fractures (Cooper & Wietlisbach 2014). Only one review on the same topic was performed in 2008. Thus, the purpose of our systematic review was to determine the available evidence on the outcomes of rehabilitation after single or multiple second to fifth metacarpal fractures sustained by adult human participants between the ages of 20 and 59 in terms of physical outcomes, disability and health-related quality of life.

## Methods

Our systematic review was registered with PROSPERO (CRD42019132620). The review protocol can be accessed at https://www.researchgate.net/profile/Monique-Keller-2.

### Eligibility criteria

Experimental study designs (randomised controlled trials [RCTs]), quasi-experimental, cohort studies and case–control studies from January 2008 to September 2018, with a language restriction of English, were included. Studies undertaken before 2008 were not included because a literature review had been performed up to 2008 (Toemen & Midgley 2010). Eligible studies met the following inclusion criteria: adult human participants older than 20 years and younger than 59 years of age. Those younger than 20 years were not included because of skeletal immaturity (De Sanctis et al. 2014), and very few individuals sustain metacarpal fractures after the age of 59; thus, no participants older than 59 years were included (Nakashian et al. 2012). Studies that report on post-surgical and conservative hand rehabilitation interventions include functional and/or non-functional exercises, other hand rehabilitation modalities/treatments/exercises and home education (could include advice, home education and home exercises [HEs]). Studies measuring outcomes which included, but were not limited to, hand function, health-related quality of life, disability, digital range of motion (ROM), grip strength and fine motor dexterity were included. Studies investigating thumb metacarpal fractures, associated tendon injury, infections, nerve injury or pre-existing osteoarthritis or rheumatoid arthritis were excluded, as were studies investigating second to fifth metacarpal fractures with a concurrent fracture of the phalangeal bones, carpal bones, distal radius and ulna.

A comparison was made according to the fracture site and amongst the varieties of hand rehabilitation programmes used: hand therapy modalities, exercises, immobilisation and home education, after surgical and conservative management. All control-intervention forms were included, and no limitations were applied.

### Information sources and search strategy

Databases and electronic platforms were searched with the assistance of an information scientist. The keywords used during the search on CINAHL are presented in Table 1.

**TABLE 1:** Database search keywords.

Search	Search string
#1	Database: CINAHL ([Boxer* or metacarpal*] n2 fracture*) and (Exercise* or program* or protocol* or ‘functional rehab*’ or rehab* or advise* or advice* or educate* or splint* or immobilise* or physiotherapy* or ‘physical therapy*’ or ‘occupational therapy*’ or outcome*)

Note: The search was limited to January 2008 to September 2018, and also limited to English.

* , indicates boolean modifiers.

Reference lists of included full-text articles were screened for potential inclusion of further eligible studies by two independent reviewers. The Internet, with the help of Google and Google Scholar, was searched for additional grey literature. The results from the searched databases are presented in Table 2.

**TABLE 2:** Databases searched and results.

Database	Number of records identified
Academic Search Ultimate	95
African-Wide Information	2
CAB Abstracts	34
CINAHL	42
Google Scholar	10
Health Source: Consumer Edition	18
Health Source: Nursing/Academic Edition	5
Scopus 21 (which indexes EMBASE)	409
MasterFILE Premier	1
MEDLINE (with full text)	220
SPORTDiscus	8
Web of Science Core Collection 21	171
Total	1015

### Study selection

All sources found during the search of the databases were subsequently imported into Endnote® (Clarivate Analytics, United States of America). Duplicates were removed. The remaining records were independently screened by two reviewers against the inclusion criteria by using the titles and abstracts. Assessment for eligibility of all remaining sources was independently performed after full-text articles were obtained for the articles included for data extraction. The inter-rater reliability of the two reviewers was high at 0.80, and a third reviewer was not needed.

### Data extraction process

The reviewers independently extracted the data with the use of an adapted Cochrane data extraction document. The adapted Cochrane document was piloted on three other studies to ensure accuracy and consistency between reviewers. No changes were made after the pilot study, and one study from the pilot study was included in the final data extraction. The information that was obtained during data extraction included: study design, demographics of participants, participant numbers, participant’s characteristics, fracture type, level and finger, randomisation settings and procedures, interventions, hand rehabilitation programmes, comparisons, controls, outcome measures, sampling details, statistical tests used and results as can be seen in Appendix Table 1-A1.

### Methodological quality appraisal of included studies

The methodological quality of all included sources was assessed with the Joanna Briggs Institute (JBI) critical appraisal checklist to assess the risk of bias (Tufanaru et al. 2017).

### Measuring the strength of the body of evidence

The grading of recommendations, assessment, development and evaluations (GRADE) method was used to test the strength of the body of evidence. The level of evidence that was accepted was of high and moderate certainty, where low and very low certainty level was documented (Oxman 2004).

### Data synthesis

The final total of three studies was included. A meta-analysis was not deemed possible because of the limited number of studies included for data extraction. A summary of the findings is, therefore, presented in Appendix Table 1-A2 and presented descriptively in the results section.

### Ethical considerations

Our study was approved by the Health Sciences Research Ethics Committee of the University of the Free State (UFS-HSD2019/0046/2602).

## Results

**FIGURE 1:**
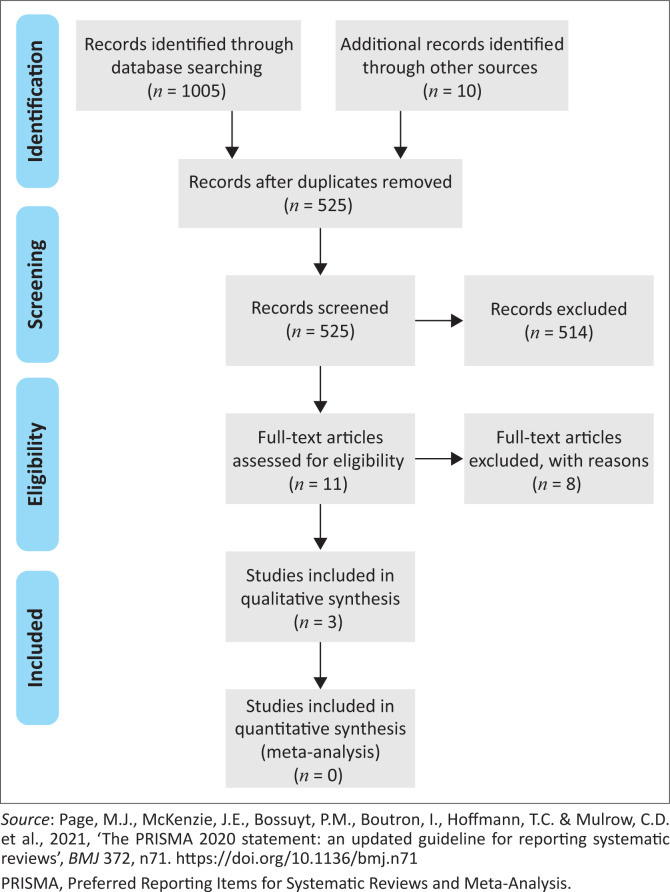
Preferred Reporting Items for Systematic Reviews and Meta-Analysis flow diagram.

The database searches generated a total of 1005 initial hits, with 10 additional sources from a Google Scholar search, and 490 duplicates were removed from the initial total of 1015 records. The Preferred Reporting Items for Systematic Reviews and Meta-Analysis (PRISMA) flow diagram (Figure 1) presents how the sources were handled through the identification, screening, eligibility and inclusion phases.

Reasons for the exclusion of 514 articles by the reviewers were: languages other than English (68), paediatric sources with participants younger than 18 (37), thumb metacarpal fractures (11), tendon injuries with an associated metacarpal fracture (15), studies on animals with metacarpal fractures (88) and only surgical intervention, with no rehabilitation, for metacarpal fractures (295). Full-text versions of the remaining 11 articles were obtained and assessed for eligibility. A further eight full-text articles were excluded with detailed reasons for exclusion in Appendix Table 1-A2. The three studies included in the review were a RCT by Gülke et al. (2018) and two observational studies by Gamble et al. (2015) and Al-Qattan (2008). The RCT had a high GRADE rating because of the study design, clear reporting and no serious inconsistencies and study limitations. The remaining two included articles scored a low grading because they both were observational studies with no evidence of strong associations in the results.

### Methodological quality of the included studies

Results revealed that the RCT study had a medium risk of bias where more than one criteria were not met (Gülke et al. 2018), one observational study had a moderate risk of bias because of unclear reporting of more than one criteria (Gamble et al. 2015) and one a high risk of bias because of more than one criteria not being met (Al-Qattan 2008). A detailed description of the included three articles will now be given.

### Description of included studies

The first observational study by Gamble et al. (2015) included 162 individuals who sustained fifth metacarpal (neck, shaft, base) fractures. The management included buddy strapping of the fourth and fifth fingers, together with issuing a handout detailing information about the fracture, guidance on early mobilisation and the natural history of the injury. No hand therapy was administered, but a handout information sheet on early mobilisation guided the rehabilitation at home. The handout information sheet advised that the buddy strapping will allow early movement, the hand should be moved and the buddy strapping should be taken off after 1 week. Participants were cautioned that the lifting of heavy objects might be painful for 6 to 8 weeks (Gamble et al. 2015).

The outcomes included client satisfaction on a four-point Likert scale, function of the upper limb assessed with the Quick Disabilities of the Arm, Shoulder and Hand (QuickDASH) questionnaire and health outcomes assessed with the EQ-5D. A postal questionnaire was utilised and a follow-up telephone call at a mean follow-up period of 21.6 months (SD = 1.9) with no other follow-up appointments. Out of the 167 individuals included in the cohort, 98 individuals (59%) responded, indicating a 31% attrition rate. A total of 79 individuals (80.6%) was very satisfied with the outcome of their fracture management, EQ-5D had a median health index score of 0.87 (interquartile range [IQR] 0.74–1.00) and hand function measured with the QuickDASH had a median score of 2.3 (IQR 0–6.8). There was a significant correlation between age and EQ-5D (*r* = −0.38, *p* ˂ 0.001) and for the QuickDASH (*r* = 0.313, *p* = 0.002). No association between gender or fracture location with the EQ-5D or QuickDASH was found. No difference could be found in EQ-5D or QuickDASH with individuals with or without a fracture. Gamble et al. (2015) advocated a ‘self-care’ management for isolated fifth metacarpal fractures. Although the information leaflet provides advice for early mobilisation, the implementation and progression of early mobilisation and ‘self-care’ remain unclear. A retrospective study design, high attrition rate, lack of robust statistical analysis and subjective assessment of outcome measures over the telephone affect the generalisability and transferability of the results.

In the second observational study, a prospective study, by Al-Qattan (2008), adult participants between the ages of 20 and 50 years were included. All 42 individuals with 54 single and multiple spiral or long oblique metacarpal shaft fractures of the long fingers were followed up after conservative treatment. Conservative treatment included a palmar wrist orthotic with the fingers left free to move, followed by immediate active and passive mobilisation of all the affected fingers with no formal physiotherapy (PT). Outcomes included: fracture healing, extensor lag at the metacarpophalangeal joint (MCPJ), total active motion (TAM) of the MCPJ and interphalangeal joints measured with 260° possible range per digit, grip strength with a dynamometer and time before returning to work. All the outcomes except grip strength (from week 6 onwards) were measured at 2 and 6 weeks, 3 months, 6 months, 9 months and 1 year. Callus formation was radiologically visible at 6 weeks (Al-Qattan 2008).

The second week follow-up included, 54 individuals’ outcomes measurement results, which were: mean extensor MCPJ lag of 26° in all fingers, TAM range mean of 234° (220° – 250°) and grip strength not assessed because of pain. Six weeks’ follow-up outcome results were measured for 54 individuals: a mean MCPJ extensor lag of 19° in all fingers, TAM range mean of 241° (230° – 255°), grip strength measurements were 60% of the contralateral hand. Only five individuals were measured during the 1 year follow-up session: no MCPJ extensor lag was present, TAM range of 260° in all fingers and grip strength was 94% (89% – 96% range) compared with the uninjured hand. Of the 54 individuals, 35 were office workers or students and they all went back to their vocations between 2 and 6 weeks. There were seven manual workers and their return to work was between 6 and 8 weeks, post-injury. A high attrition rate was observed where 25 individuals’ outcomes were measured at 6 weeks and only 5 at the final follow-up. A concern exists that outcome measures for pain, hand function and disability were not assessed, as these are measurements required to determine the success of a conservative and immediate mobilisation hand rehabilitation programme ­(Al-Qattan 2008).

In the third included study, Gülke et al. (2018) conducted a prospective cohort RCT on 60 participants who sustained a single diaphyseal or metaphyseal second to fifth metacarpal fracture. The RCT aimed to determine the effectiveness of a traditional PT programme compared with a developed HE programme after an open reduction internal fixation (ORIF) surgical management. The participants were divided into two groups, a PT and HE group with the use of standardised, controlled block randomisation. After a 2-week splinting period for both groups, the intervention and control group programme commenced. The PT group received 12 units of 30-min PT over 6 weeks (between 3 and 8 weeks after injury). The therapists administering treatment for the PT group were instructed to provide exercises that could be performed at home. No controlled PT programme existed. The HE programme group was instructed to perform the exercises three times a day, four to six exercises per session and for a period of 20 min – 30 min. For the HE programme, the first week after immobilisation included: scar treatment for five 5 min – 10 min, a chamomile bath for 5 min, decongestive massage for 5 min, three times 10 repetitions of active fist making and three times 10 repetitions of crocodile metacarpal exercises. The second week after the immobilisation included: repeat exercises from week 1 and add three times 10 repetitions of an upper limb stretching exercise (‘steal and hide cherries’); for week 3 and 4: stop the decongestive massage, active fist making and crocodile metacarpal exercises. Add three sets of 10 repetitions of rolling a pen up in fingers, flexing from the distal interphalangeal joints to the MCPJ, and 10 repetitions of opening pegs with unaffected and affected fingers. For weeks 5 and 6, the pen roll-ups and peg exercises were stopped, the previous exercises were continued and three sets of 10 repetitions of ball squeezes were added. The follow-up assessments at 2 weeks post-surgery demonstrated a severe loss of digital ROM in both groups. The ROM measured at 3 months improved to 245° and 256° TAM for the PT and HE group, respectively, from a normal digit ROM of 270°. The TAM ROM for the HE group (256°) was significantly higher than the (245°) TAM for the PT group (*p* = 0.013). The grip strength measurements improved from 6 to 12 weeks for the PT group from 68% to 91% and for the HE group, from 71% to 93% compared with the uninjured hand. Mean DASH score at 6 weeks for the PT group was 30 and for the HE group 25. The mean DASH score at 12 weeks was 16 for the PT group and 14 for the HE group (*p* ˂ 0.0001). The findings suggested that a well-developed HE programme after post-surgical management for second to fifth (non-thumb) metacarpal fractures can be as effective as traditional PT rehabilitation (Gülke et al. 2018).

From these three studies, the following could be included in a post-surgical intervention programme:

- A handout information sheet on early mobilisation and rehabilitation at home (Gamble et al. 2015).
- A palmar wrist orthotic with the fingers left free to move, followed by immediate active and passive self-mobilisation of all the affected fingers with no formal PT (Al-Qattan 2008).
- Two-week splinting followed by a developed HB exercise programme of free active MCPJ exercises, strengthening, neurodynamic exercises, stretching exercises and scar treatment (Gülke et al. 2018).

## Discussion

High-level evidence (Level 1b) from the RCT included in our systematic review indicates that a well-developed and instructed HE programme after a surgical management for second to fifth metacarpal fractures is as effective or even more effective than traditional PT (Gülke et al. 2018). The variability and quality of the other included studies make it difficult to draw definite conclusions on the best hand rehabilitation programme after a surgical and conservative management for second to fifth metacarpal fractures.

Only studies from 2008 were included. A systematic review that included a wider age range and covered all literature up to 2008 is now presented and compared with the results of our review. The evidence-based treatment pathway for second to fifth metacarpal fractures was compiled by Midgley and Toemen (2010). A systematic literature review was conducted and included published sources up to 2008. The intervention entailed a period of splinting and early active mobilisation of all the unaffected joints that were not splinted. This was applied for all types of fractures. The treatment for metacarpal fractures was as follows: conservative or surgical management with K-wires was used for extra- and intra-articular fractures. At 4 weeks after the injury, light function and wrist exercises were commenced. In the instance where fracture management was performed surgically with an ORIF, light function was commenced at 2 weeks. After the conservative or K-wire management of MCPJ shaft fracture treatment, exercises were started at 3 weeks and included wrist and MCPJ active movement to regain full ROM. At 4 weeks, light function was commenced. Where the metacarpal shaft fracture was managed surgically with an ORIF, light function was introduced at 2 weeks. For neck and head metacarpal fractures that were managed either conservatively or with K-wire fixation, light function was introduced at 4 weeks. For neck and head metacarpal fractures that were managed with ORIF, light function was introduced earlier at 2 weeks. Strengthening was only commenced at 6 weeks.

Midgley and Toemen (2010) tested the developed evidence-based pathway, in an observational study, on a sample of 50 participants. The included participants’ second to fifth metacarpal fractures were either managed surgically or conservatively (Midgley & Toemen 2010). Phalangeal fractures and thumb metacarpal fractures were excluded. Treatment sessions delivered by a therapist specialising in hand therapy included: fabrication of a splint, treatment and an information leaflet. Outcomes were measured based on telephonic interviews with 23 participants carried out at a period of 10–24 weeks after sustaining the injury. Splint compliance was 47%, with no complications, 72% patients had no pain, all participants who were employed had returned to work, full hand function was restored in 92% of participants and the service satisfaction was 8/10, with an average of three therapy sessions administered. A small sample size, low splinting compliance, omission of outcomes such as ROM, disability and grip strength, lack of standardised outcome measures used and assessments by using a telephonic interview, however, compromise the generalisability of the results.

The findings from Midgley and Toemen’s (2010) and Toemen and Midgley (2010) studies suggest the following:

- A period of splinting and early active mobilisation of all the unaffected joints; encouragement of graded light function, self-mobilising exercises and lastly strengthening exercises could be of benefit to be included post-surgery in the rehabilitation of second to fifth metacarpal fractures.

No definite conclusion can be drawn about the best hand rehabilitation programme after surgical or conservative management for second to fifth metacarpal fractures based on the previous review by Midgley and Toemen (2010) and our review. The possible reasons are different management approaches used amongst participating healthcare practitioners, the limited number of high-quality studies, the presence of heterogeneity and the strict inclusion criteria, ages and years, in our review. There are, however, studies describing early active mobilisation in various age groups that may improve a post-surgical and conservative programme (Debnath et al. 2004; Feehan & Bassett 2004; Khan & Giddins 2015; Muller et al. 2003; Retrouvey, Morzycki & Wang 2018; Wong & Higgins 2017; Yalizis et al. 2017).

This is however contradicting the question asked by Retrouvey et al. (2018) on whether we are overtreating hand fractures. Based on this controversy and research question they conducted a national survey in Canada. The cross-sectional survey was completed by 113 plastic surgeons, where 50% of the participants had more than 15 years of experience. Seventy-three per cent of surgeons prefer splinting and early active ROM, where 21% would instead immobilise the fracture after splinting and no early mobilisation. An interesting finding was that years of experience and practice did not influence the decision between surgical or non-surgical approaches, but the surgeons with more years of practice did not refer their patients to hand therapists (Retrouvey et al. 2018). Perhaps the reason for the paucity in hand rehabilitation programmes found in this review could be because of a lack of referral to a hand therapist.

Feehan and Bassett (2004) conducted a systematic literature review to assess the effectiveness of early mobilisation on function and fracture healing for individuals who sustained extra-articular hand fractures. Studies included those investigating the comparisons between a complete immobilisation of joints proximal and distal to the fracture and an early mobilisation commenced before 21 days of one or both joints proximal and distal to the fracture. The authors concluded that no level I or II evidence could be found to support or refute early motion ≤ 21 days of the joints surrounding the fracture. Interestingly, as in our review, the authors could only give a narrative description of the results because of the limited number and heterogeneity amongst sources.

Further evidence for early mobilisation was found in a study by Debnath et al. (2004) who conducted a prospective study on 17 participants after fifth metacarpal shaft fractures. Management included reduction and hand-based casting to immobilise the fracture for 1 month where the wrist and MCPJs were left free for immediate mobilisation. The fractures of three participants lost the reduction (15° – 20° of angulation), but after a 6-month follow-up, all fractures healed fully with no functional deficits. Khan and Giddins (2015) also used early mobilisation after conservative management for 25 participants who sustained spiral metacarpal fractures. The outcome measures of grip strength and ROM ranged from good to excellent and all fractures healed after 6 months. Another prospective RCT included 35 participants who had sustained a boxer’s fracture with an angulation of 15° – 70° (Muller et al. 2003). A 3-week immobilisation period was compared with a pressure dressing applied for 1 week with immediate immobilisation. No statistically significant difference was found for pain, ROM and satisfaction outcomes at a 3-month follow-up. Van Aaken et al. (2016) in a high-grade evidence-based, randomized, multicentre trial on 68 participants who sustained boxer’s fracture with ≤ 70° angulation and no rotational deformities compared immediate active wrist and finger mobilisation to forearm wrist POP immobilisation. Both groups had good outcomes. The only statistically significant difference was days off work. On an average, the participants of the early mobilisation groups returned to their occupation 11 days earlier (*p* = 0.03).

After a surgical management, early mobilisation also seems to be successful. An observational study on 16 Australian Rules football players who sustained second to fifth metacarpal fractures and managed surgically with reduction and fixation (Yalizis et al. 2017) showed a return to professional play in 2 weeks with a soft glove and a protective dressing to protect the sutures with good hand function in 12 participants. Fracture union was seen in all participants at 6 weeks. These studies give a reasonable assurance that early mobilisation after surgical and conservative management is safe in shaft and boxer’s fractures, which are minimally displaced (Wong & Higgins 2017).

### Recommendations

Based on our review the standardised outcome measurements used in assessing outcomes improve the transferability of the HE programme and have the added benefit of potentially saving resources for stakeholders, medical staff and injured individuals across the world (Gülke et al. 2018). This is especially beneficial in countries where the incidence of metacarpal fractures is high because of violence and the resources and hand rehabilitation expertise are limited. However, generalisation is difficult as countries with limited resources do not always have the instrumentation at their disposal to perform ORIF. Future high-quality research is recommended where an HE programme is implemented not only for individuals with second to fifth metacarpal fractures that were managed surgically with an ORIF but also for conservative and K-wire management, to inform clinical practice when surgical management is not indicated and in countries where surgical interventions are not always possible because of limited resources.

### Limitations

The lack of controlled studies and heterogeneity of the included studies prevented the conduction of a systematic review of efficacy, which would be the design of choice to determine the highest level of evidence in this field.

## Conclusion

Level 1b evidence is available from an RCT indicating that a well-designed HE programme is the best and most effective rehabilitation programme after surgical management for second to fifth metacarpal fractures, where the hand function and digital TAM are statistically and significantly higher than the traditional PT programme group (Gülke et al. 2018). Some evidence is available on early active mobilisation for minimally displaced, conservatively managed, spiral and long oblique metacarpal shaft fractures (Al-Qattan 2008) and fifth metacarpal fractures (Gamble et al. 2015).

## Appendix 1

**TABLE 1-A1:** Summary of findings.

Authors	Sampling strategy	Description of rehab/exercise/ splint modality	Follow-up periods	Profession delivering intervention	How is treatment delivered	Outcome measures used (for each outcome)	Results on outcomes 1st assessment (Copy for each outcome)	Results on outcomes 2nd and more assessments (Copy for each outcome)	Grade
Al-Qattan (2008) (No control group)	Purposive sampling (all patients with spiral or long oblique metacarpal shaft fractures of the fingers who were treated conservatively with a palmar wrist splint between 2003 and 2006 were studied prospectively).	No formal PT Active and passive exercises of all finger joints.	2 weeks, 6 weeks, 3 months, 6 months, 9 months, 1 year (patients included with a minimum of 6 weeks follow up)	Doctor (surgical)	Conservative management (face to face)	TAM Grip strength	*N* = 54 2 weeks TAM = lag of 26° (mean range - 234°) Grip strength = difficult to assess 6 weeks TAM = mean lag of 19° (mean range - 241°) Grip strength = 60% *N* = 36 3 months TAM = mean lag is 15° (mean range = 253°) Grip strength = 74%	*N* = 25 6 month TAM = 5° lag in 2 (mean range - 260°) Grip strength = 81% *N* = 11 9 months TAM = 260° Grip strength = 90% *N* = 5 TAM = 260° Grip strength = 94%	Low
Gamble et al. (2015) (No control group)	Purposive sampling (The patient cohort was collated from a search of the Emergency Department’s information system, Omnis, that identified patients directly discharged from the Emergency Department with a fracture of the fifth metacarpal.	Functional bracing - neighbour strapping (buddy) Information leaflet - outlining diagnosis, advice for early mobilisation and a helpline contact number	No follow up (but questionnaire sent out a minimum 1-year post-intervention)	Emergency medicine doctor or emergency nurse practitioner	Bracing Information leaflet	Satisfaction Likert scale EQ-5D Quick Dash	Post 1 year: 80.6% were very satisfied Median EQ-5D = 0.87 Median Quick Dash = 2.3	No other outcomes measured	Low
Gülke et al. (2018)	Standardised controlled block randomization.	Custom made Functional dorsal orthotic device (Light Cast) fixated with an elastic wrap. MPJs = 70 flexion; PIP, DIPJs free movement. Removed post 2 weeks. Physio teaching exercises that can be done at home After week 8, exercises carried out independently	Group 1: 2 weeks post-surgery: 12 units of PT over 6 weeks (week 3–8) Week 8–12: Independent (Group 1 & 2) Reassessment at week 12	Hand surgeon Physio	Individual physio session (30 mins twice per week)	ROM (neutral zero methods) Week 2: MPJ: 42.5° PIPJ: 88.3° DIPJ: 89.1° Jamar Dynamometer DASH	ROM Week 6 MPJ: 61.7° PIPJ and DIPJ - increased a little Grip strength Week 6 68% DASH Week 6 Score 30	ROM Week 12: MPJ: 73.3° Grip strength Week 12 91% DASH Week 12 Score 16	High
Gülke et al. (2018) Control group	Standardised controlled block randomization.	Custom made Functional dorsal orthotic device (Light Cast) fixated with an elastic wrap. MPJs = 70 flexion; PIP, DIPJs free movement. Removed post 2 weeks. Exercise booklet with pictures, individual exercises. Questions answered by hand surgeon after reading.	Group 2: 2 weeks post-surgery: independent exercises (week 3 - 8) Week 8–12: Independent (Group 1 & 2) Reassessment at week 12	Hand surgeon Self-management	Exercise booklet	ROM (neutral zero method) Week 2 MPJ: 46.5° PIPJ: 86.8° DIPJ: 89.8° Jamar Dynamometer DASH Documenting exercise	ROM Week 6 MPJ: 68.5° PIPJ and DIPJ - increased a little Grip strength Week 6 71% DASH Week 6 Score 25	ROM Week 12: MPJ: 73.3° Grip strength Week 12 93% DASH Week 12 Score 14	

## Appendix 2

**TABLE 1-A2:** Reasons for exclusion.

Authors	Inclusion Criteria				Exclusion Criteria				Comments
	Study design: Experi, quasi, RCT, cohort, case-control (2008 –2018)	Participants 20 – 59 years	Intervention: post-surgical, hand rehab, home ed, immob	Outcomes: Function and pre-functional	Presence of tendon or nerve injury; pre-existing arthritis	Other # (phalanges, carpals, radius and ulna)	Thumb metacarpal #	Presence of infections	
Al-Qattan	✓ (prospective/cohort)✕	✓	✓	✓	✕	✕	✕	✕	
Cepni et al.	✓ (prospective)	✕	✓	✓	✕	✕	✕	✕	Excluded due to not meeting full inclusion criteria (18 year patients)
Gamble et al.	✓ (retrospective)	✓	✓	✓	✕	✕	✕	✕	
Gulabi et al.	✓ (retrospective)	✕ (age range 10 – 66 years)	-	-	-	-	-	-	Excluded due to not meeting full inclusion criteria
Gulke et al.	✓	✓	✓	✓	✕	✕	✕	✕	
Khan & Giddins	✓ (prospective)	✕ (age range 17 – 60 years)	-	-	-	-	-	-	Excluded due to not meeting full inclusion criteria
Klibanoff & Potter	?	-	-	-	-	-	-	-	Excluded - only abstract given
MacDonald et al.	✓ (prospective)	✕ (age range 11 – 54 years)	-	-	-	-	-	-	Excluded due to not meeting full inclusion criteria
Midgley & Toeman	✓ (prospective)	✕ (age greater than 16 years)	-	-	-	-	-	-	Excluded due to not meeting full inclusion criteria
Moon et al.	✓ (retrospective)	✕ (age range, 16 –73 years)	-	-	-	-	-	-	Excluded due to not meeting full inclusion criteria
Strub et al.	✓ (experimental)	✕ (group B range from 21 – 70 years)	-	-	-	-	-	-	Excluded due to not meeting full inclusion criteria
